# On Gastrodynia

**Published:** 1868

**Authors:** W. H. Day

**Affiliations:** Physician to the Margaret Street Infirmary for Consumption and Diseases of the Chest


On Gastrodynia. By W. H. Day, M. D., M. R. C. P., Physician to the Margaret Street Infirmary for Consumption and Diseases of the Chest.

There are few more painful affections, and very few more difficult to cure, than that peculiar functional derangement of the stomach known as gastrodynia, or gastric neuralgia. We know that neuralgia may arise from functional or organic causes. The nervous system may be sufficiently deranged to pervert the function of sensation, varying in degree from mere tingling and numness to positive and unendurable pain. Angina pectoric, colic, gastrodynia, irritable testicle and uterus are all familiar examples of altered sensation and of agonizing pain in the sympathetic system, or in one or other of its nervous ganglia. When the nerves of special sense are affected, we have tinnitus aurium, muscæ volitantes, and alterations of smell and taste. Looking to organic change as one of the causes of neuralgia, we may have lesions of the brain or spinal cord, and the pressure of tumors and spiculæ of bone, irritating the nervous centres. There is pain down in the left arm from disease of the heart, pain in the right shoulder from structural change in the liver, and sciatica sometimes springs from disease of the hip-joint. A cancerous growth of the stomach, in its early stages, will cause precisely the same train of symptoms as gastrodynia, arising from poverty of blood and defective assimilation. Gastrodynia occurs under a variety of circumstances, sometimes selecting for its victims the apparently strong and plethoric—at others the temperate and intemperate; but the nervous, the anxious, and the anæmic, and those of feeble constitutional vigor, are most prone to the affection. Those who indulge largely in food and stimulants, and take little exercise, are often the subjects of it. In most cases, the circulation will be found at fault, and the nervous system imperfectly nourished. It is met with in persons of sedentary habits, and is very common in rural districts among the poor, who drink strong and hot tea, and scarcely ever taste animal food. I regard tea-drinking as a most common cause, and in men I have several times traced the misery to drinking coffee after a full dinner. The lower class in the north of England, who eat oat-cake, suffer greatly from gastrodynia. These are the most obstinate cases to treat, and they will not yield to treatment till the diet is improved. This complaint is usually met with in middle life, and in women more frequently than in men. The pain of gastrodynia is peculiar and almost characteristic; it is referred to the situation of the solar plexus, to a spot not larger than a shilling in some cases,* whilst in others it occupies the whole epigastric region, shooting through to the back, beneath one or more scapulæ. When the pain is severe, and the patient has fasted some hours (and he will avoid eating to escape the torture that ensues), the stomach becomes enfeebled, and there is much flatulence. The over-distended stomach will of itself produce the phenomena; but in addition, it will press up the diaphragm, and so mechanically interfere with the proper rythmic action of the heart, causing palpitation, dyspnœa, faintness, and even syncope. After these attacks (for the pain often comes on in paroxysms of severity), patients frequently complain of superficial soreness and pain, so that they cannot bear the weight of their clothes. Some cases are relieved by taking food (probably from the temporary increased secretion of gastric juice); other cases are greatly aggravated by it, and there is positive torture until the food is rejected by vomiting, or it has passed through the pyloric aperture. Brodie has said that, since gastrodynia most frequently occurs in atonic cases, and often depends upon flatulence, any diffusible stimulant, giving temporary tone to the stomach, and causing its muscular fibres to contract and expel flatus, will give temporary relief. In many cases, there is neuralgic headache, as a sympathetic action through the vagus. When this is present, it greatly assists our diagnosis of the cause of the epigastric suffering. The pain is far greater than the ordinary pain of dyspepsia; it is not the burning pain of gastric ulcer, nor the sharp and lancinating pain of cancer; it is a pure neuralgia—a dull, heavy, gnawing pain; and whatever exhausts the nervous system, as grief, anxiety, fear, bad living, etc., favors the complaint. It is now and then attended by hypochondriacal symptoms, and depression of spirits. Anæmia is a most fruitful source of this affection, and tubercular disease of the lungs is a frequent accompaniment. As in gastric ulcer, constipation and amenorrhœa are often present, and for the same reasons—viz., the small amount of food which is taken, and the bloodless condition of the patient.

* These are by far the most difficult cases to treat; they resist one remedy after another; I have found preparations of iron and counter-irritation most applicable to this class of cases.

*Treatment.*—Under this head I shall speak, separately, of the chief remedies which have been tried from time to time. If the digestive organs are much deranged, and there is acidity, with a thickly-coated tongue, and especially if there is pyrosis, then magnesia, soda, and bismuth or lithia are of service. These may not be enough to effect a cure of themselves, but they put the stomach in better order, and fit it for other remedies. I do not wish to underrate these medicinal agents, but I have not found any great benefit follow their employment. Their prolonged use weakens the digestive functions by neutralizing the acids of the acidulated pepsine, which is the solvent for the nitrogenous or flesh-producing ailments, and there can be no doubt that many cases of habitual indigestion are owing to the frequent and indiscriminate use of these alkalies. When given, I prefer them in combination with sedatives, as prussic acid and very small doses of morphia. If the tongue is reddish at the tip, with prominent papillæ, and there are signs of gastric irritation, bismuth is a remedy of great value. It will be of benefit only when there is excess of secretion on the part of the stomach. When the the tongue is clean and pale, it does positive harm. Sometimes small doses give relief; but if the case is obstinate, large doses (from ten to fifteen grains) will prove effectual where smaller doses would fail. I prescribe the liquor bismuthi (Schacht), one drachm, with the same quantity of syrup of ginger, and two minims of the dilute hydrocyanic acid, on an empty stomach, three times a day before meals.*

Preparations of iron are especially to be recommended, from the unhealthiness of the blood, and the general debility which is usually present. I have seen most benefit derived from the citrate of iron and strychnine, in doses of four grains in an ounce of water, three times a day, taken after food. This is a most valuable medicine when the tongue is clean and pallid, and it is particularly indicated if the pain is at all paroxysmal. Occasionally in this disorder, and in other debilitated states of the system, this preparation of iron and strychnine does not agree; and lately two cases have come under my notice in which headache, sickness and great nervous agitation followed each dose. The same unpleasant symptoms have resulted when the remedy was pre-

* The trochisci bismuthi contains two grains of bismuth in each lozenge, and several may be taken with advantage during the day.

scribed in doses of two grains. I need scarcely say, under these circumstances, the medicine must be given up directly. Sometimes the ammonia-citrate of iron will effect a cure after this has failed. At the same time that these remedies are given, counter-irritation over the epigastrium is often of much service.* In one case, attended with great anæmia and extreme feebleness of digestive power, five minims of the tincture of the sesquichloride of iron, given three times a day in an ounce of water, effected a cure when all other remedies that were tried, so far from relieving the pain, tended materially to increase it. The nitrate and oxide of silver are not always to be relied on, but they occasionally come to our assistance when other remedies have ended in disappointment. The most obstinate cases have recovered after other medicines had been given in vain. They are most suitable when the tongue is clean and pale. The oxide has rendered me the most service.

The purified oxide of manganese has been recommended in doses varying from five to fifteen grains, three times a day, on an empty stomach. It may be given in the cases for which we prescribe bismuth, and I am compelled to say I have seen benefit derived from it, though it does not prevent a return of the complaint, like the preparations of iron, when they can be tolerated. The mineral acids, which I prefer to give alone with water, are very useful in many cases of gastric pain, and where the action of the liver is sluggish. They may be given when the urine is turpid and high-colored; and, in fact, as the symptoms improve, the urine will soon become clear. I give the acids in full doses —five minims of the dilute nitric acid, and ten minims of the dilute hydrochloric acid, in an ounce of water—an hour before meals. These acids now and then greatly aggravate the pain of gastrodynia. If the bowels are costive, a pill of Barbadoes aloes, capsicum and quinine, given daily before dinner, will hasten the cure. It is not so much to the remedy which the physician prescribes, that he must attribute his success, as it is to the nice discrimination of all the points that are presented to his view; how he may attack the strongest, yet protect the weakest; how best meet a case having many prominent symptoms, and combat those first which are the most important. On the judicious selection

* The emp. piscis co., to which three or four grains of tartarized antimony is added, answers well; the size of the plaster should be about three inches square.

of these for management, the issue may altogether depend.

I have selected a few cases which best illustrate the plan of treatment recommended:

CASE 1. *Gastrodynia; treatment with oxide of silver.*—Mrs. S——, aged forty-two (May, 1862), has suffered at intervals during the past thirteen years from pain at the pit of the stomach, and nothing afforded relief for any length of time. She now complains of constant aching at the epigastrium, accompanied with great flatulence, palpitation and lowness of spirits. Has headache, coming on usually at night. The tongue is pale and swelled. All the symptoms are aggravated by eating. Pulse 80, very weak. She was ordered the mineral acids, with chloric ether and infusion of colomba, three times a day.

May 28th. No improvement; pain and flatulence very great; tongue a little furred. She was ordered three grains of the compound rhubarb pill and one grain of capsicum before dinner daily, and the following draught three times a day: Aromatic spirit of ammonia, half a drachm; chloric ether ten minims; tincture of ginger and compound tincture of lavender, of each haif a drachm, to an ounce and a half of water.

June 16th. No benefit. Ordered one-twelfth of a grain of morphia and three grains of bismuth, in a pill, to be taken three times a day.

Aug. 3d. She has been much better; but the pain and distensions are very trying.

20th. Being still about the same, I prescribed the following pill three times a day, and all other medicine was omitted; Powdered rhubarb, half a drachm; oxide of silver, six grains; muriate of morphia, a grain and a half; confection of roses in sufficient quantity to make twenty pills.

April 1st, 1863. She now complains of slight return of the symptoms, after entire freedom from the headache, palpitation and pain during the winter months. She resumed the medicine, and lost the symptoms entirely in a week. In January, 1866, she had had no return, had gained flesh, and was perfectly well in health.

CASE 2. *Gastrodynia; treatment with mineral acids and steel.* G. A——, agel twenty-five, unmarried, a seamstress by occupation (April 13th, 1861). She complains of a heavy dragging pain at the pit of the stomach, always aggravated by taking food. She never vomits her food; the pulse is small and quick; the face pasty and pallid; there is amenorrhœa and constipation, palpitation, and a very perceptible anæmic bruit over the base of the heart. She was ordered the mineral acids, with tincture of nux vomica, three times a day, before food, and a pill twice a week, consisting of compound colocynth pill, extract of hyoscymus, and capsicum.

25th. She has derived some benefit, but is very weak; the bowels now act well. Ten minims of the tincture of the muriate of iron were added to each dose of the mixture.

29th. She has entirely lost the pain. To continue the mixture for a month.

Case 3. M. S——, aged fifty-one, married (April 4th, 1861), has suffered from gastrodynia for many months, and can get no relief. She is a sallow-looking woman, and has cataract in both eyes. She drinks a great deal of tea, and says she cannot afford meat. The tongue is large, pale and clean; the pulse very weak; there is constipation and great flatulence. Similar treatment to that in case No. 2 was adopted, and she expressed herself as quite well on the 25th.

Case 4. *Gastrodynia; treatment by iron and strychnine.* F. M——, aged twenty-three, single, (April 20, 1864), by occupation a servant; a pale, melancholy-looking girl. She has constant pain in the stomach, and is afraid to eat. There is headache, with a feeble pulse, and great nervous debility. Tongue, clean, red, and fissured. Has been taking "thick white medicine" for a month without relief. She was ordered the following pill three times a day: Citrate of iron and strychnine, three grains; quinine, one grain; confection of roses in sufficient quantity. She was quite well in a month.

In Jan., 1867, she applied again, with similar symptoms, and stated that at the close of last year she had spitting of blood. A physical examination of the chest revealed tubercle in its early stage of developement, and I should, therefore dispair of affording any lasting benefit.

Case 5. *Severe gastrodynia, complicated with hepatic congestion.* R. P——, aged fifty-three, (July, 1863), married, a gamekeeper, complained of great pain in the stomach, and inability to digest food. He was so worn and emaciated that I strongly suspected malignant disease. Has been unable to follow his employment for six months, and no medicine has done him any good. The urine is acid, scanty, and high-colored; the tongue is thickly coated; there is great flatulence, pyrosis, and cardiac uneasiness. He was ordered bismuth and magnesia, with dilute hydrocyanic acid, and chloric ether.

Aug. 1st. There was no material improvement. Tenderness existed over the region of the liver, and he had a very sallow aspect. In addition to the mixture, he was ordered one grain of blue pill, and four grains of the extract of taraxacum, every night for a week.

6th. He was much improved in every respect. The infusion of columba was added to the bismuth draught.

10. The tongue was clean, and the urine clear. There was no pyrosis, and the pain had much abated. Ordered the bismuth to be omitted, and one teaspoonful of the following mixture to be taken in a wineglassful of water three times a day: Citrate of iron and strichnine, two drachms; water, four ounces.

Sept. 5th. He was quite well, and able to resume his work, having gained flesh and strength, and being entirely free from pain.

In July, 1864, there was a return of the symptoms, which gave way in a week to the mercurial and steel. There was in this case some hepatic congestion, not very apparent on examination, which accounted for the symptoms so speedily yielding when the blue pill was prescribed.

The following case is another example of the advantage of similar treatment:

Case 6. *Severe gastrodynia; hepatic congestion.* E. M——, aged twenty-eight (May, 1862,) a clergyman, suffered severely for upwards of two years with similar symptoms to those of Case No. 5, and all medicines failed to give him relief till he took a little blue pill. He was exceedingly thin, and had no appetite, and a feeble pulse. I prescribed bismuth, magnesia, and colomba before lunch and before dinner, and the following pills twice a week at bedtime: Mercury pill, twelve grains; extract of hyoscyamus, ten grains; compound rhubarb pill, two scruples: divide into twelve pills, two to be taken for a dose. He was quite well in a month, but could never tolerate any preparation of steel.

About this time another clergyman consulted me with very severe gastrodynia, and after trying several remedies to no purpose (including bismuth in a mixture,) the following pill three times a day completely cured him: Subcarbonate of bismuth, one drachm; powdered rhubarb, one scruple; muriate of morphia, two grains; powdered capsicum, ten grains: in thirty pills.

Notwithstanding the best-directed treatment, cases now and then go unrelieved, and we are obliged to admit the inefficacy of medicine to deal with them. The following cases are examples:

CASE 9. *Severe gastrodynia; no relief from treatment.* H. W——, aged twenty-seven, (April 22, 1864,) a cook, fat and flabby; had fever fourteen years ago, and lost "small bones from the ear;" very deaf ever since. She has had gastrodynia for the past ten years at intervals; had synovial inflammation of the right knee-joint a year and a half ago. The tongue is large, pale, and fissured; the gums white, and teeth large and irregular. There is occasional headache on one side. The bowels are regular; there is flatulence, unattended by proysis. The following are among the remedies unsuccessfully prescribed:

Dec. 31st, 1863. Subcarbonate of bismuth and carbonate of magnesia, of each two grains; muriate of morphia, one grain; to be made into twelve powders. To take one in a little milk, three times a day.

Jan. 12th. Great relief followed, till

March 22nd, when the pain returned as violently as ever. A liniment was rubbed over the epigastrium night and morning, and a pill of steel, quinine, and rhubarb, taken three times a day.

April 7th. Pain is not mitigated. The oxide of silver, with rhubarb, and very small doses of morphia and capsicum, were prescribed.

10th.—No relief whatever. Ordered the purified black oxide of manganese, in doses of ten grains, three times a day, and counter-irritation over the epigastrum.

22d.—The pain subsided for four days, and then returned as acutely as before. The citrate of iron and strychnine was now given, and this afforded relief for a week. I did not see her again till

July 13th, when the pain was again most severe. She was admitted into the Cambridge Hospital, and came out much relieved. Some time after I heard that her suffering was so great that she had to give up her situation as a servant.

On referring to my note-book, I find that a nobleman consulted me for atonic neuralgia of the stomach on the 29th of April, 1861. He was sallow, and had a worn and exsanguine aspect. The suffering had been of ten years' duration, coming on invariably at night, and depriving him of rest and sleep. He would get up and walk for hours in his room. He had always been most temperate in his habits, and never smoked. Two of his family died of malignant disease of the stomach. The tongue was tolerably clean, the bowels regular, and the urine clear. There was no vomiting, no flatulence, no headache—only the severe gnawing, incessant pain. His diet consisted of cocoa, milk, and arrowroot. He described himself as a complete "vegetarian," and said traveling from place to place did him more good than all the physic he had ever taken. He had consulted the most eminent men in London and Paris without deriving the least benefit; on the contrary, medicines increased his sufferings by acting as strong irritants. I once prescribed for him, and added greatly to his misery; and this had been the case with others who had been consulted. A month ago (June, 1867,) he told me that he now ate pastry, and almost any kind of diet, and never felt the slightest pain whatever. His general health and strength had vastly improved.

